# Corneal Keratocyte Density and Corneal Nerves Are Reduced in Patients With Severe Obesity and Improve After Bariatric Surgery

**DOI:** 10.1167/iovs.62.1.20

**Published:** 2021-01-21

**Authors:** Zohaib Iqbal, Alise Kalteniece, Maryam Ferdousi, Safwaan Adam, Luca D'Onofrio, Jan H. Ho, Anoop Prasanna Rao, Shaishav Dhage, Shazli Azmi, Yifen Liu, Rachelle Donn, Rayaz A. Malik, Handrean Soran

**Affiliations:** 1 Faculty of Biology, Medicine and Health, University of Manchester, Manchester, United Kingdom; 2 Cardiovascular Trials Unit, Manchester University NHS Foundation Trust, Manchester, United Kingdom; 3 Department of Medicine, Weill Cornell Medicine–Qatar, Doha, Qatar; 4 Department of Experimental Medicine, Sapienza University of Rome, Rome, Italy

**Keywords:** bariatric surgery, obesity, keratocytes, corneal nerves, corneal confocal microscopy

## Abstract

**Purpose:**

Obesity is associated with peripheral neuropathy, which bariatric surgery may ameliorate. The aim of this study was to assess whether corneal confocal microscopy can show a change in corneal nerve morphology and keratocyte density in subjects with severe obesity after bariatric surgery.

**Methods:**

Twenty obese patients with diabetes (*n* = 13) and without diabetes (*n* = 7) underwent assessment of hemoglobin A1c (HbA1c), lipids, IL-6, highly sensitive C-reactive protein (hsCRP), and corneal confocal microscopy before and 12 months after bariatric surgery. Corneal nerve fiber density (CNFD), corneal nerve branch density (CNBD), corneal nerve fiber length (CNFL), and keratocyte density (KD) from the anterior, middle, and posterior stroma were quantified. Twenty-two controls underwent assessment at baseline only.

**Results:**

CNFL (*P* < 0.001), CNBD (*P* < 0.05), and anterior (*P* < 0.001), middle (*P* < 0.001), and posterior (*P* < 0.001) keratocyte densities were significantly lower in obese patients compared to controls, and anterior keratocyte density (AKD) correlated with CNFL. Twelve months after bariatric surgery, there were significant improvements in body mass index (BMI; *P* < 0.001), HDL cholesterol (*P* < 0.05), hsCRP (*P* < 0.001), and IL-6 (*P* < 0.01). There were significant increases in CNFD (*P* < 0.05), CNBD (*P* < 0.05), CNFL (*P* < 0.05), and anterior (*P* < 0.05) and middle (*P* < 0.001) keratocyte densities. The increase in AKD correlated with a decrease in BMI (*r* = –0.55, *P* < 0.05) and triglycerides (*r* = –0.85, *P* < 0.001). There were no significant correlations between the change in keratocyte densities and corneal nerve fiber or other neuropathy measures.

**Conclusions:**

Corneal confocal microscopy demonstrates early small fiber damage and reduced keratocyte density in obese patients. Bariatric surgery leads to weight reduction and improvement in lipids and inflammation and an improvement in keratocyte density and corneal nerve regeneration.

Obesity mediates metabolic derangement and activation of inflammatory pathways^1^ which improve after bariatric surgery, some independently of weight loss.^2^ Obesity-associated small fiber neuropathy^3^ is thought to occur as a result of mitochondrial dysfunction, oxidative stress, extracellular protein glycation, and inflammation.^4^ Smith et al.^5^ showed that obesity and dyslipidemia were associated with a reduction in intraepidermal nerve fiber density. Studies have shown that bariatric surgery is associated with an improvement in macrovascular outcomes^6^ but to a lesser extent microvascular complications.^7^ With regard to neuropathy, Miras et al.^8^ showed no significant changes in nerve conduction, whereas Muller-Stich et al.^9^ reported a significant improvement in neuropathic symptoms and neuropathy disability score after bariatric surgery.

Corneal confocal microscopy (CCM) is a rapid, non-invasive ophthalmic technique that can accurately quantify small fiber degeneration and regeneration in patients with diabetes^10^ and other peripheral neuropathies.^11^^–^^13^ CCM also allows in vivo visualization of other corneal layers, including the epithelium, stromal keratocytes, and endothelium.^14^ Corneal keratocytes are essentially fibroblasts of mesenchymal origin contained within the stroma.^15^ Corneal keratocyte density is greatest in the anterior stroma and decreases progressively toward the posterior stroma.^16^ Keratocytes are normally quiescent but become activated after corneal injury to promote fibrosis and repair in the cornea.^17^ Nakamura et al.^18^ demonstrated an increase in alpha-smooth muscle actin, a marker of keratocyte activation, after photorefractive keratectomy in rabbits. Zieske et al.^19^ showed that epithelial debridement leads to keratocyte mitosis. Penetrating injuries to the cornea result in increased epithelial cell production of transforming growth factor-beta (TGF-β) and activation of stromal keratocytes.^20^ We have previously reported a reduction in keratocyte density in the anterior (AKD), middle (MKD), and posterior (PKD) stroma and suggested that it may be associated with corneal nerve loss in diabetic neuropathy.^21^ Although studies by Frueh et al.^22^ and Quadrado et al.^23^ have shown no change in KD, Bitirgen et al.^24^ demonstrated a reduction in KD and corneal nerves in patients with diabetes. The aim of this study was to evaluate if keratocyte density and corneal nerve morphology are altered in patients with severe obesity and whether they change after bariatric surgery.

## Methods

### Study Subjects

Twenty patients with severe obesity undergoing bariatric surgery at a tier-4 bariatric surgery service in Manchester, UK, and 22 age-matched healthy controls were recruited. All patients underwent Roux-en-Y gastric bypass, except two who underwent single anastomosis gastric bypass. Obese patients were seen before and 12 months after bariatric surgery, and the controls underwent a single assessment at baseline. Patients were excluded if they had a history of malignancy, history of corneal trauma or disorders including dry eyes, previous eye surgery, laser therapy, chronic renal or hepatic impairment, previous radiotherapy or chemotherapy, or other cause of peripheral neuropathy. Informed consent was obtained from each participant before recruitment. This research adhered to the tenets of the Declaration of Helsinki. All participants provided written informed consent. The study was approved by the Greater Manchester Research Ethics Committee (REC No. 11/NW/0731, IRAS ID: 85208)

### Clinical and Metabolic Assessment

At baseline, all participants underwent a medical questionnaire and measurement of height, weight, and waist circumference. Venous blood was taken between 9:00 AM and 11:00 AM (after an overnight fast) for analysis of hemoglobin A1c (HbA1c), IL-6, highly sensitive C-reactive protein (hsCRP), and total lipid profile. Total cholesterol, triglycerides, and HDL cholesterol in serum were determined using a Cobas Mira auto-analyzer (Roche Diagnostics, Basel, Switzerland) with calibrator, control, and reagents (Randox Laboratories, Crumlin, UK). hsCRP was measured using an in-house ELISA and CRP antibodies, calibrators, and controls from Abcam (Cambridge, UK); IL-6 was determined using the DuoSet ELISA Development System (DY206; R&D Systems, Minneapolis, MN, USA).

### Neuropathy Assessment

Clinical assessment of neuropathy was undertaken using the modified neuropathy disability score (NDS)^25^ and neuropathy symptom profile (NSP)^25^ questionnaires. The vibration perception threshold was measured using a Horwell Neurothesiometer (Scientific Laboratory Supplies, Wilford, UK) with an average of three readings from each foot. The TSA 2 Quantitative NeuroSensory Analyzer (Medoc Ltd., Ramat-Yishai, Israel) was used to quantify cold (CT) and warm (WT) perception thresholds on the dorsum of the foot. Cardiac autonomic function was quantified by evaluating deep breathing heart rate variability (DB-HRV) measured by the ANX 3.0 Autonomic Nervous System monitoring device (ANSAR Medical Technologies, Inc., Philadelphia, PA, USA).^26^

### Corneal Confocal Microscopy

All participants underwent CCM assessment using a laser scanning confocal microscope (Heidelberg Retina Tomograph 3 with Rostock Cornea Module; Heidelberg Engineering, Heidelberg, Germany) according to our previously published protocol.^27^ The first high-quality image of the stroma inferior to Bowman's layer represented the anterior keratocyte layer, an image superior to Descemet's membrane represented the posterior keratocyte layer, and an intermediate image between the anterior and posterior layers represented the middle keratocyte layer. CCMetrics (University of Manchester) was used to manually count keratocytes, and their density was expressed as the number of cells per square millimeter of stroma (cells/mm^2^). For quantification of corneal nerves, six images from the central cornea were selected using our established protocol.^28^ Three corneal nerve parameters were quantified: corneal nerve fiber density (CNFD), the total number of main nerves per square millimeter (no./mm^2^); corneal nerve branch density (CNBD), the number of primary nerve branches per square millimeter (no./mm^2^); and corneal nerve fiber length (CNFL), the total length of main nerves and nerve branches per square millimeter (mm/mm^2^). Ophthalmic technicians were blinded when carrying out image analysis.

### Statistical Analysis

SPSS Statistics 22 (IBM Corporation, Armonk, NY, USA) was used to carry out statistical analysis. The Shapiro–Wilk test was used to determine whether the distribution of the data was parametric or non-parametric. Parametric data were expressed as mean ± SD, and non-parametric data were expressed as median ± interquartile range (IQR). Parametric data were compared using Student's *t*-test, paired for related samples and unpaired for unrelated ones. The Mann–Whitney *U* test was used for non-parametric unpaired variables and the Wilcoxon signed-rank test for paired data. *P* < 0.05 was considered significant. Correlations were carried out using Pearson's test for parametric data and Spearman's rank test for non-parametric data. Percentage change was calculated by dividing the difference between baseline and 12 months by the baseline. Percentage difference was calculated by dividing the difference between controls and baseline by the controls.

### Power Analysis

Based on data from a previous study,^29^ a sample size of 17 participants was calculated to have 80% power to detect a within-patient change of 4 fibers/mm^2^ in CNFD. This assumes a 5% significance level and a standard deviation of within-subject differences of 5.5.

## Results

### Comparisons Among Baseline Parameters in Obese Patients and Controls

Twenty patients with obesity were recruited and compared to 22 age- and sex-matched healthy controls at baseline. Body mass index (BMI; *P* < 0.001) and weight (*P* < 0.001) were significantly higher in obese patients compared to controls. Total cholesterol (*P* < 0.001) and LDL cholesterol (*p* < 0.05) were lower in obese patients compared to controls, with no significant difference in triglycerides (*P* = 0.08). HbA1c was significantly higher in the obese patients (*P* < 0.001) compared to controls (Table 1). Thirteen obese patients had type 2 diabetes (HbA1c, 52 ± 13.7 mmol/mol) and seven did not (HbA1c, 36.4 ± 2.7 mmol/mol). Anthropometric measurements and measures of neuropathy, CCM, and keratocyte densities were comparable between obese patients with and without diabetes (Supplementary Table S1).

**Table 1.** Clinical, Metabolic, Neuropathy, and CCM Parameters in Patients with Obesity Compared to Controls Before Bariatric Surgery

	Controls	Obese	Percentage Difference,^*^ %	*P*
Number	22	20	—	—
Type 2 diabetes	0	13	—	—
Age, y	54.0 ± 9.4	48.8 ± 8.3	—	0.81
Anthropometric**^†^**				
Height, cm	167.3 ± 10.2	168.5 ± 10.4	+0.7	0.78
Weight, kg	76.4 ± 14.8	142.96 ± 7.4	+85.8	<0.001
BMI, kg/m^2^	28.0 ± 5.1	49.5 ± 9.2	+76.8	<0.001
Laboratory data**^‡^**				
HbA1c, mmol/mol	33.0 ± 8.6	42 (37–56)	+27	<0.001
Total cholesterol, mmol/L	5.65 ± 0.26	4.7 ± 1.31	–16.8	<0.001
Triglycerides, mmol/L	1.0 (0.75–1.75)	1.81 (0.9–2.29)	+81	0.08
LDL cholesterol, mmol/L	3.26 ± 0.73	2.5 ± 1.08	–23.3	<0.05
Keratocytes, no./mm^2^				
AKD	625 (625–727)	491.09 ± 97.68	–21.4	<0.001
MKD	406.79 ± 45.85	318.12 ± 61.7	–21.7	<0.001
PKD	382.4 ± 52.08	321.87 ± 69.2	–17.9	<0.01
Corneal nerves				
CNFD, no./mm^2^	29.84 ± 5.84	26.8 ± 5.12	–10.2	0.20
CNBD, no./mm^2^	96.69 ± 22.15	74.2 ± 36.45	–23.3	<0.05
CNFL, mm/mm^2^	29.03 ± 4.31	20 (18–23)	–31.1	<0.001
Neuropathy assessments				
NDS (score out of 10)	0 ± 0	2.8 ± 2.6		<0.001
NSP (score out of 38)	0.19 ± 0.5	1.5 (0–8.75)	+689	<0.001
VPT, V	6.0 ± 4.3	13.1 ± 7.2	+118.3	<0.001
CT, °C	26.5 ± 4.5	24.5 ± 5.6	–7.5	0.16
WT, °C	37.8 ± 4.3	41.0 ± 4.3	+6.3	<0.05
DB-HRV, beats/min	24.1 ± 13.7	21.2 ± 8.1	–12.0	0.28

Data are presented as mean ± SD for parametric variables and median (interquartile range) for non-parametric variables.

* Percentage difference relates to difference between obese patients and controls.

† Waist circumference was not measured in controls.

‡ HDL and IL-6 were not measured in controls.

### Neuropathy

NDS (*P* < 0.001), NSP (*P* < 0.001), vibration perception threshold (VPT; *P* < 0.001), and WT (*P* < 0.05) were higher, but DB-HRV (*P* = 0.28) did not differ between obese patients and controls.

### Corneal Confocal Microscopy

CNFL (*P* < 0.001) and CNBD (*P* < 0.05) were significantly lower for obese patients, but CNFD (*P* = 0.20) did not differ between obese patients and controls. Anterior (491.09 ± 97.68 vs. 616.47 ± 124.45; *P* < 0.001), middle (318.12 ± 61.7 vs. 406.79 ± 45.85; *P* < 0.001), and posterior (321.87 ± 69.30 vs. 382.4 ± 52.08; *P* < 0.05) keratocyte densities were significantly lower in obese patients compared to controls (Figs. 2 and 3).

### Post-Bariatric Surgery

See Table 2^3^.

**Table 2.** Clinical, Laboratory, Neuropathy, and CCM Measures Before and 12 Months After Bariatric Surgery

	Baseline (*n* = 20)	12 Months	Percentage Change, %	*P*
Anthropometric measures				
Weight, kg	142.8 ± 7.4	97.4 ± 24.4	–31.8	<0.001
Waist circumference, cm	140.3 ± 22.7	106.4 ± 23.3	–24.2	<0.001
BMI, kg/m^2^	49.5 ± 9.2	34.1 ± 7.1	–31.1	<0.001
Laboratory measures				
HbA1c, mmol/mol	42 (37–56)	34.4 ± 3.13	–18.1	<0.05
Total cholesterol, mmol/L	4.7 ± 1.31	4.40 ± 0.79	–6.4	0.67
Triglycerides, mmol/L	1.81 (0.9–2.29)	1.21 (0.75–1.56)	–33.1	<0.05
LDL cholesterol, mmol/L	2.5 ± 1.08	2.72 ± 0.71	+8.8	0.19
HDL cholesterol, mmol/L	0.92 ± 0.23	1.17 ± 0.18	+27.2	<0.05
hsCRP, mg/L	3.99 (2.2–9.9)	1.6 ± 1.6	–60	<0.001
IL-6, pg/mL	5.26 (2.54–7.89)	2.1 (1.02–5.66)	–60	0.002
Keratocytes, no./mm^2^				
AKD	491.09 ± 97.68	616 (494–625)	+25.4	<0.05
MKD	318.12 ± 61.70	343.09 ± 55.13	+7.8	<0.001
	321.87 ± 69.30	381.39 (312–403)	+18.5	0.15
Corneal nerves				
CNFD, no./mm^2^	26.8 ± 5.12	31.1 ± 5.03	+16.0	<0.05
CNBD, no./mm^2^	74.2 ± 36.45	90.8 ± 35.33	+22.4	<0.05
CNFL, mm/mm^2^	20.0 (18–23)	22.3 ± 4.52	+11.5	<0.05
Neuropathy measures				
NDS (score out of 10)	2.5 ± 2.14	0.5 (0.5–4.75)	–80	0.26
NSP (score out of 38)	1.5 (0–8.75)	1 (0–8)	–33.3	0.14
VPT, V	13.1 ± 7.27	10 (8–16)	–23.7	0.28
CT, °C	24.5 ± 5.6	26.1 ± 3.5	+6.5	0.27
WT, °C	41.0 ± 4.12	41.9 ± 3.47	+2.2	0.60
DB-HRV, beats/min	21.2 ± 8.1	23.3 ± 10.9	+9.9	0.57

Data are presented as mean ± SD for parametric variables and median (interquartile range) for non-parametric variables.

**Table 3.** Correlations Among Percent Changes (%Δ) in Anterior, Middle, and Posterior Keratocyte Densities With Percent Change in Clinical, Metabolic, and Corneal Nerve Parameters

	Correlations		
Parameter	%Δ AKD	%Δ MKD	%Δ PKD
BMI	*r* = –0.55, *P* < 0.05	*r* = –0.31, *P* = 0.25	*r* = –0.07, *P* = 0.78
HbA1c	*r* = –0.06, *P* = 0.86	*r* = –0.15, *P* = 0.24	*r* = –0.23, *P* = 0.46
Triglycerides	*r* = –0.85, *P* < 0.001	*r* = –0.74, *P* < 0.01	*r* = 0.20, *P* = 0.57
hsCRP	*r* = –0.02, *P* =0.94	*r* = –0.2, *P* = 0.52	*r* = 0.38, *P* = 0.40
IL-6	*r* = 0.08, *P* = 0.81	*r* = –0.11, *P* = 0.75	*r* = 0.29, *P* = 0.41
CNFD	*r* = –0.11, *P* = 0.77	*r* = 0.25, *P* = 0.46	*r* = 0.05, *P* = 0.88
CNBD	*r* = –0.25, *P* = 0.49	*r* = –0.22, *P* = 0.50	*r* = 0.31, *P* = 0.39
CNFL	*r* = 0.49, *P* = 0.13	*r* = 0.21, *P* *=* 0.52	*r* = 0.51, *P* = 0.11

#### Clinical and Laboratory

Twelve months after bariatric surgery there were significant reductions in weight (*P* < 0.001), BMI (*P* < 0.001), and waist circumference (*P* < 0.001). There was a significant reduction in triglycerides (*P* < 0.05) and increase in HDL (*P* < 0.05) but no change in total cholesterol (*P* = 0.67) or LDL cholesterol (*p* = 0.19). There were significant reductions in hsCRP (*P* < 0.001) and IL-6 (*P* < 0.01).

#### Neuropathy Measures

**Figure 1.**
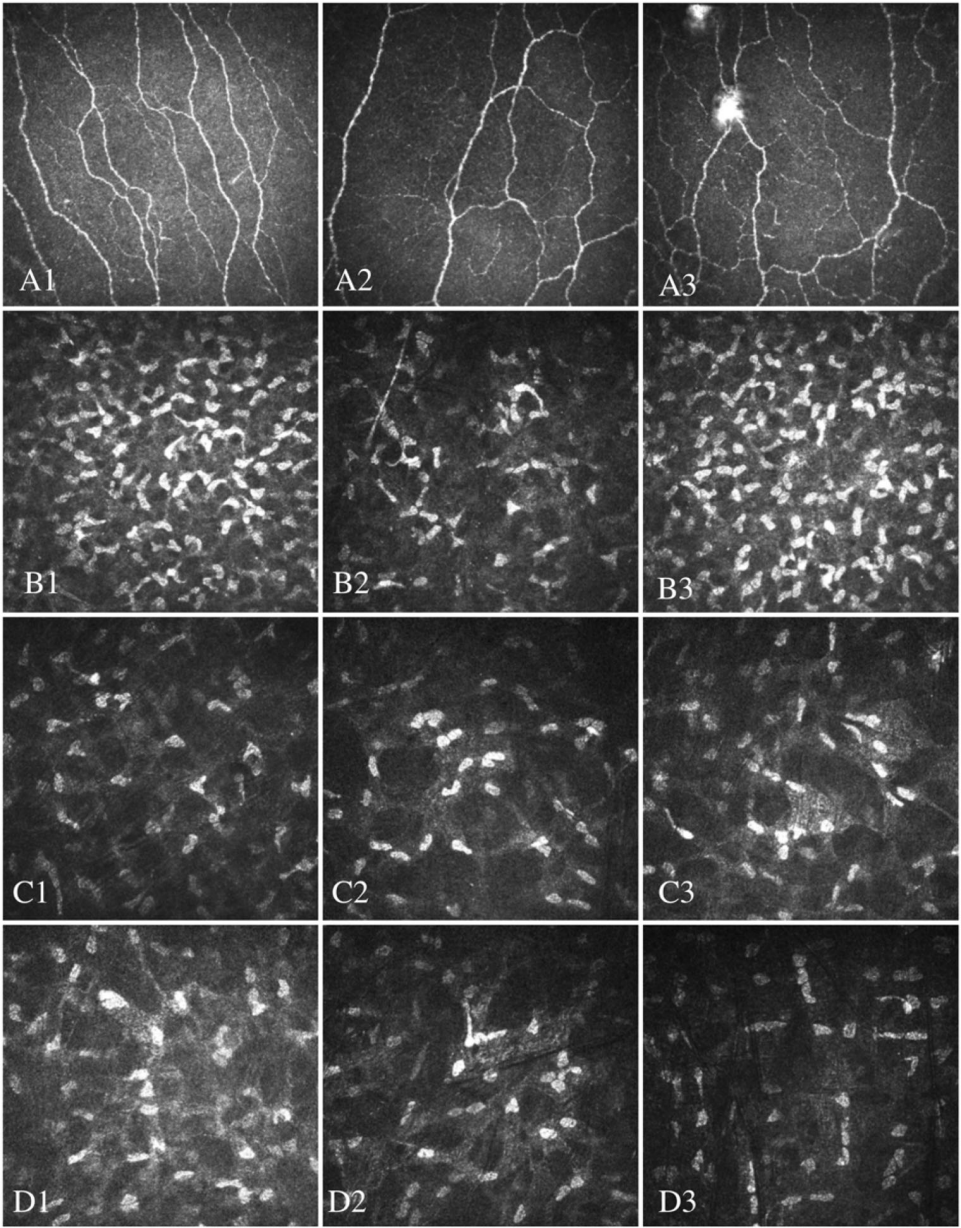
CCM images of sub-basal nerve plexus (**A1**–**A3**) and anterior (**B1**–**B3**), middle (**C1**–**C3**), and posterior (**D1**–**D3**) stromal keratocytes in healthy controls (**A1**–**D1**) and obese patients before (**A2**–**D2**) and after (**A3**–**D3**) bariatric surgery.

There was no significant change in the NSP (*P* = 0.14), NDS (*P* = 0.26), VPT (*P* = 0.28), CT (*P* = 0.27), WT (*P* = 0.60), or DB-HRV (*P* = 0.57) after bariatric surgery in obese patients (Table 2). However, there were significant increases in CNFD (*P* < 0.05), CNBD (*P* < 0.05), and CNFL (*p*<0.05), as well as anterior (*P* < 0.05) and middle (*P* < 0.001) keratocyte densities (Table 2, Fig. 1^2^,^3^).

**Figure 2.**
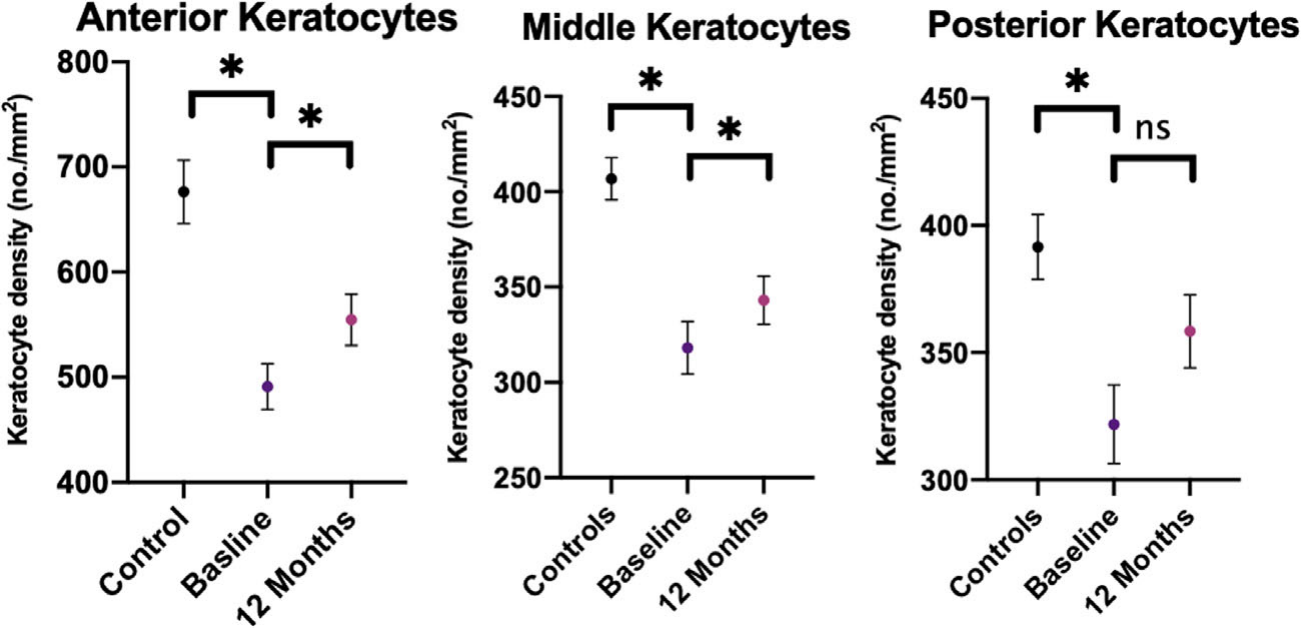
Error plots showing mean and SEM of anterior, middle, and posterior keratocyte densities in controls and obese patients at baseline and 12 months. ^*^P < 0.05.

**Figure 3.**
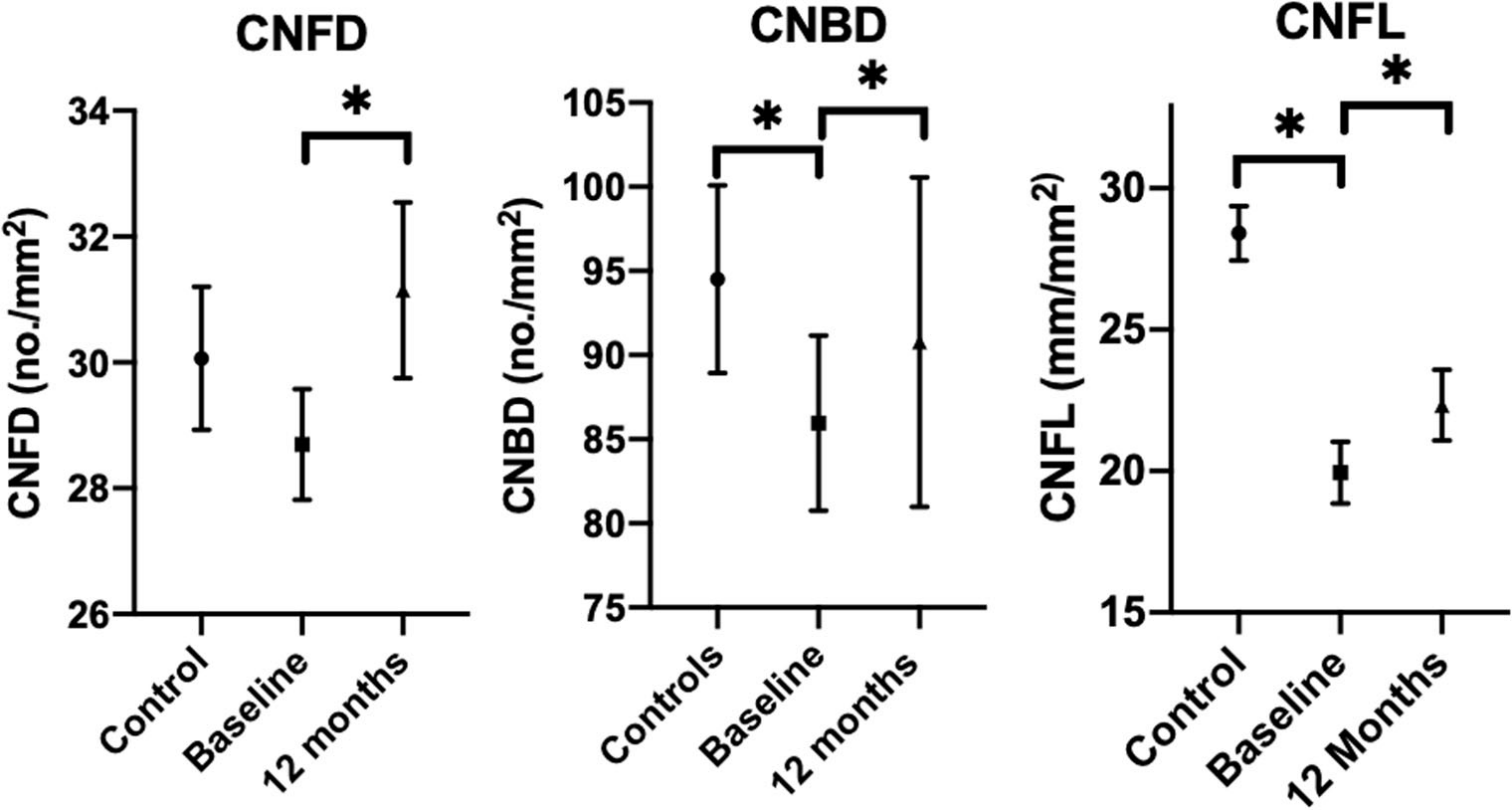
Error plots showing mean (SEM) of CNFD, CNBD, and CNFL in controls and obese patients at baseline and 12 months after bariatric surgery. ^*^P <0.05.

#### Correlations

**Figure 4.**
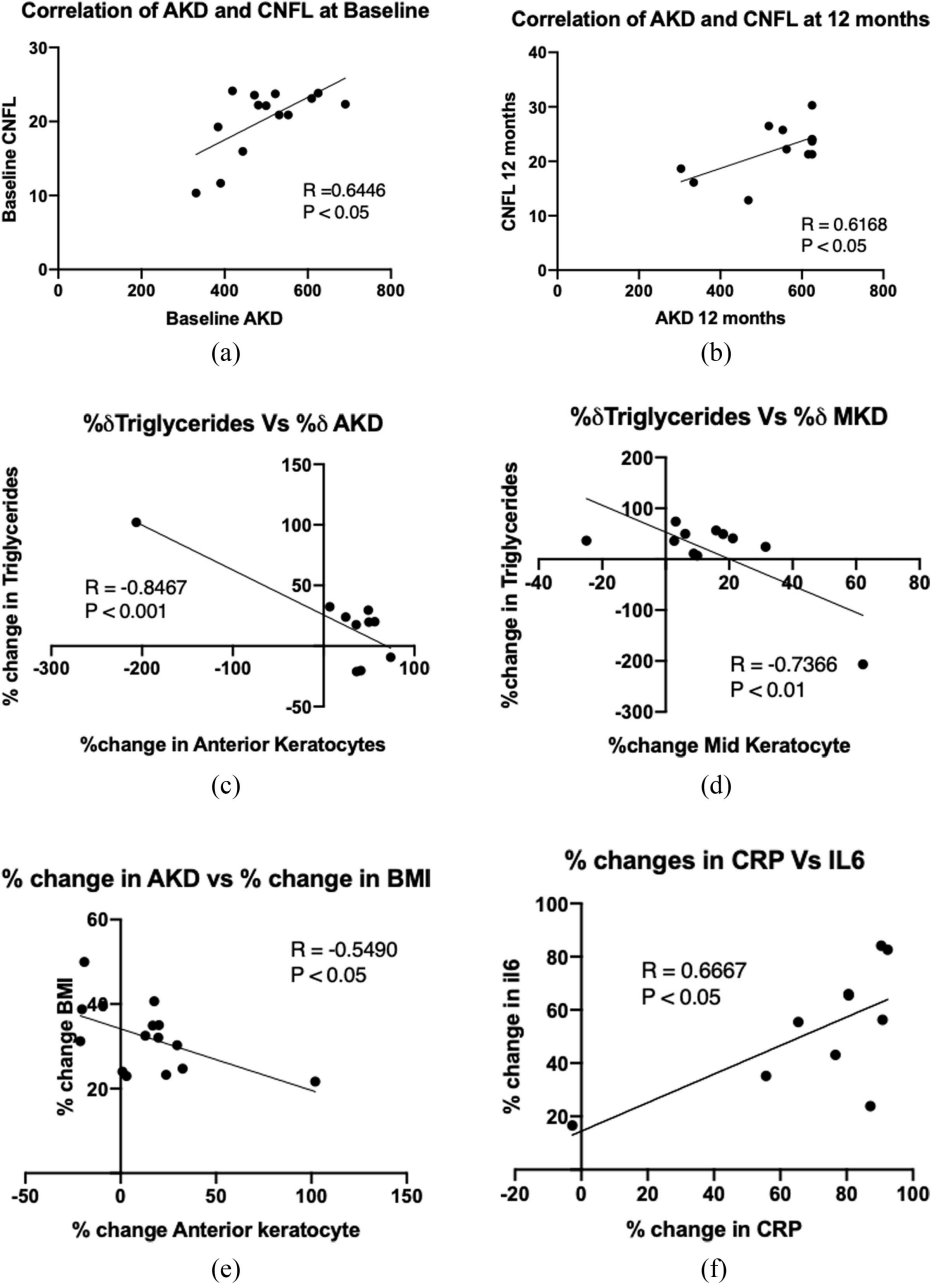
Correlation plots among clinical, metabolic, and inflammatory markers with CCM markers.

At baseline, AKD correlated with CNFL (*r* = 0.64, *P* < 0.05), and this correlation (*r* = 0.61, *P* < 0.05) was maintained at 12 months. There were no significant correlations among AKD, MKD, PKD, hsCRP, and IL-6 or any corneal nerve parameter. The percentage increase in AKD correlated with the percentage decrease in BMI (*r* = –0.5, *P* < 0.05) and triglycerides (*r* = –0.8, *P* < 0.001) (Fig. 4), and the percentage increase in MKD correlated with the percentage decrease in triglycerides (*r* = –0.74, *P* < 0.01) (Fig. 4). The percentage change in hsCRP correlated with the percentage change in IL-6 (*r* = 0.7, *P* < 0.05) (Table 3).

## Discussion

To our knowledge, this is the first study to show a reduction in corneal keratocyte densities and corneal nerves in subjects with obesity which improved 12 months after bariatric surgery. Bariatric surgery resulted in significant improvements in weight, BMI, and waist circumference and a 72% remission of type 2 diabetes.^30^ Corneal keratocytes lie in close proximity to corneal nerves within the stroma.^16^ Lambiase et al.^31^ showed that corneal keratocytes produce neurotrophins similar to dermal keratocytes, which play an important role in nerve fiber maintenance and repair in the skin.^32^ Yam et al.^33^ reported a dose-dependent relationship between activated stromal keratocytes and nerve growth. The anterior keratocytes are in close proximity to the sub-basal nerve plexus, and we found a significant correlation between anterior keratocyte density and CNFL which was maintained 12 months after bariatric surgery, but no significant association between MKD and PKD and corneal nerves. However, the relative increase in keratocyte density was small in magnitude and may require a larger and longer study to confirm or refute the relationship between keratocytes and corneal nerves. Interestingly changes in AKD density correlated with changes in BMI and triglycerides after bariatric surgery. Indeed, previously the laser Doppler imaging flare response, a marker of small nerve fiber function, has shown a significant inverse relationship with triglycerides.^34^ The cornea is avascular, and we cannot attribute a direct mechanistic link^35^; however, increased triglycerides and BMI are associated with incident diabetic neuropathy,^36^ and we have shown that higher triglycerides correlate with the severity of corneal nerve damage in patients with acute ischemic stroke.^37^

There was evidence of a mild subclinical neuropathy and corneal nerve loss in the patients with obesity, consistent with experimental studies showing an obesity-related neuropathy.^3^ Yorek et al.^38^ demonstrated a reduction in subepithelial corneal nerves and corneal sensitivity in obese rats, and Davidson et al.^39^ found a reduction in the corneal sub-basal plexus of rats with type 2 diabetes and obesity using corneal confocal microscopy.

After bariatric surgery, there was no improvement in neuropathic symptoms or deficits, vibration and thermal perception thresholds, or cardiac autonomic neuropathy, but there was a significant improvement in corneal nerve parameters, indicative of nerve regeneration. This is in contrast with a study showing an early improvement in the neuropathy symptom score and NDS after bariatric surgery.^9^ Indeed, a recent systematic review and meta-analysis of four studies has shown an improvement in the neuropathy symptom score and NDS, particularly in diabetic patients with a BMI < 35 following bariatric surgery.^40^ However, Miras et al.^41^ found no improvement in nerve conduction studies 12 months after Roux-en-Y bypass. Previously, we have also shown that bariatric surgery was associated with an improvement in corneal nerves and neuropathic symptoms with no change in quantitative sensory testing or nerve conduction studies after 12 months.^42^ Indeed, we and others have also demonstrated early corneal nerve fiber repair with no change in other measures of neuropathy after simultaneous pancreas and kidney transplantation^43^ and treatment with ARA 290^44^^,^ ^45^ and omega-3^45^ in patients with diabetes. These data add to the emerging evidence that CCM can identify early improvement in small nerve fibers prior to other measures of neuropathy.

This study also found elevated serum IL-6 and hsCRP, reflecting chronic low-grade inflammation in obese patients. Triglycerides, HDL, IL-6, and hsCRP improved significantly after bariatric surgery, suggesting resolution of adipose-related inflammation. Randell et al.^46^ also showed a reduction in hsCRP and ferritin 12 months after sleeve gastrectomy, and meta-analyses have confirmed a reduction in hsCRP and IL-6 after bariatric surgery.^47^ Previous studies have demonstrated an association between elevated IL-6 and hsCRP with peripheral neuropathy in diabetes^48^ and other cohorts,^49^ particularly those with painful neuropathy.^50^ However, in the present study, although circulating levels of IL-6 and hsCRP improved, this improvement did not correlate with the improvement in corneal nerve parameters.

We acknowledge that a limitation of the present study is the small cohort of subjects studied with obesity with and without diabetes. Furthermore, although the neuropathy phenotyping was detailed, we did not undertake nerve conduction studies or skin biopsy. The duration of follow-up of 12 months was relatively short.

## Conclusions

Severe obesity is associated with small fiber damage and reduced keratocyte density. Bariatric surgery leads to a significant reduction in weight and an improvement in lipids and markers of inflammation, with an increase in keratocyte density and corneal nerve regeneration. Further work is required to elucidate the underlying mechanisms for corneal nerve fiber repair.

## Supplementary Material

Supplement 1
