# Supplementary material for: Corneal Keratocyte Density and Corneal Nerves Are Reduced in Patients With Severe Obesity and Improve After Bariatric Surgery
Source: Invest Ophthalmol Vis Sci. 2021 Jan 21;62(1):20. doi: 10.1167/iovs.62.1.20 (PMC7817877; doi:10.1167/iovs.62.1.20)
Supplement: Supplement 1 [file iovs-62-1-20_s001.pdf]

Supplementary Table 1	Diabetes	No Diabetes	*p* value
**HbA1c**	52.0 ± 13.7	36.4 ± 2.7	$p < 0.05$
**Anthropometric**			
Height (cm)	170 ± 11.8	165 ± 8.3	$p = 0.35$
Weight	143 ± 29.0	128 ± 28.0	$p = 0.28$
**Keratocytes**			
AKD (no./mm^2)	506.7 ± 83	462 ± 121.0	$p = 0.32$
MKD (no./mm^2)	336 ± 62.0	283 ± 45.0	$p = 0.06$
PKD (no./mm^2)	343 ± 71.0	281 ± 46.0	$p = 0.05$
**CCM Parameters**			
CNFD (no./mm^2)	26.8 ± 5.1	31.1 ± 5.0	$p = 0.43$
CNBD (no./mm^2)	73.9 ± 40.0	73.9 (63.5 – 82.3)	$p = 0.77$
CNFL (mm/mm^2)	19.7 ± 4.5	21.4 (19.2 – 22.2)	$p = 0.84$
**Neuropathy Parameters**			
NDS (score out of 10)	1.8 ± 2.5	3.6 ± 2.7	$p = 0.93$
NSP (score out of 38)	4.2 ± 4.8	3.1 ± 4.3	$p = 0.98$
CT (°C)	25.0 (20-27)	26.4 (22-29)	$p = 0.55$
WT (°C)	42.7 (39-43)	38.6 (36-45)	$p = 0.56$
DB-HRV (beats/min)	20.1 ± 13.8	18.0 ± 7.3	$p = 0.73$

Data are presented as mean ± standard deviation for parametric variables and median (interquartile range) for non-parametric variables.
